# Microfabricated Modular Scale-Down Device for Regenerative Medicine Process Development

**DOI:** 10.1371/journal.pone.0052246

**Published:** 2012-12-19

**Authors:** Marcel Reichen, Rhys J. Macown, Nicolas Jaccard, Alexandre Super, Ludmila Ruban, Lewis D. Griffin, Farlan S. Veraitch, Nicolas Szita

**Affiliations:** 1 Department of Biochemical Engineering, University College London, London, United Kingdom; 2 Centre for Mathematics and Physics in the Life Sciences and Experimental Biology, University College London, London, United Kingdom; 3 Department of Computer Science, University College London, London, United Kingdom; University of Bristol, United Kingdom

## Abstract

The capacity of milli and micro litre bioreactors to accelerate process development has been successfully demonstrated in traditional biotechnology. However, for regenerative medicine present smaller scale culture methods cannot cope with the wide range of processing variables that need to be evaluated. Existing microfabricated culture devices, which could test different culture variables with a minimum amount of resources (e.g. expensive culture medium), are typically not designed with process development in mind. We present a novel, autoclavable, and microfabricated scale-down device designed for regenerative medicine process development. The microfabricated device contains a re-sealable culture chamber that facilitates use of standard culture protocols, creating a link with traditional small-scale culture devices for validation and scale-up studies. Further, the modular design can easily accommodate investigation of different culture substrate/extra-cellular matrix combinations. Inactivated mouse embryonic fibroblasts (iMEF) and human embryonic stem cell (hESC) colonies were successfully seeded on gelatine-coated tissue culture polystyrene (TC-PS) using standard static seeding protocols. The microfluidic chip included in the device offers precise and accurate control over the culture medium flow rate and resulting shear stresses in the device. Cells were cultured for two days with media perfused at 300 µl.h^−1^ resulting in a modelled shear stress of 1.1×10^−4^ Pa. Following perfusion, hESC colonies stained positively for different pluripotency markers and retained an undifferentiated morphology. An image processing algorithm was developed which permits quantification of co-cultured colony-forming cells from phase contrast microscope images. hESC colony sizes were quantified against the background of the feeder cells (iMEF) in less than 45 seconds for high-resolution images, which will permit real-time monitoring of culture progress in future experiments. The presented device is a first step to harness the advantages of microfluidics for regenerative medicine process development.

## Introduction

Over the last ten years, bioreactor miniaturisation for traditional biotechnology has made significant progress. What began with a proof-of-concept study [1] is now a field of its own, broadly encompassing miniaturised stirred tank, microwell format-based and microfabricated bioreactors [2], [3], [4], [5]. Favourable comparisons with larger scale bioreactors have been successfully demonstrated with bacterial, yeast and Chinese Hamster Ovary cells, and these mini- and micro-bioreactors have been operated in batch, fed-batch and chemostat mode. Automated, parallelised and instrumented, miniaturised bioreactors deliver quantitative data on the growth kinetics in real time, from culture volumes as small as 5 microlitres [6]. Several systems are now commercially available and could underpin the implementation of the Process Analytical Technologies and Quality by Design initiatives [7]; in short, bioreactor miniaturisation has changed the way early stage process development can be approached in traditional biotechnology.

In traditional mammalian cell culture applications, cells are typically adapted to grow in suspension, either freely or attached to microcarriers. However, regenerative medicine presents the bioprocessing industry with a new production challenge, in which the cells themselves are the product. While some progress has been made towards the development of microcarrier-based expansion of human embryonic stem cells (hESC) [8], early clinical trials of stem cell medicines rely on more traditional adherent culture [9], [10], [11]. To deliver a range of potential clinical applications [10], [12], [13], [14], [15] it will be necessary to reliably, safely and efficiently produce high quality cells in adherent cultures [16], [17], [18]. To optimise the numerous biological, physical and chemical factors that synergistically combine to control stem cell fate [19], a large amount of process development is necessary. Consequently, due to the high cost of media components and the slow growth rate of stem cells, it is obvious that regenerative medicine process development will benefit from a similar technology drive towards miniaturisation.

Present smaller scale culture methods limit stem cell process development. In culture flasks and dishes, the high cost of the growth factor-containing media constrains the number of experiments that can be performed. On the other hand, microwell plates, which operate with smaller amounts of media, are susceptible to well-to-well variability, medium evaporation and edge effects [20]. Additionally, all these devices typically lack instrumentation, giving a reduced understanding of the impacts of process variables. There are also problems with variations during manual processing, which can affect the phenotype of stem cells [21], [22]. Microfabricated devices show potential to overcome these issues.

A number of publications have clearly demonstrated that stem cell culture can be performed with fewer resources at a microfluidic scale [23] and different platform technology and parallelisation approaches have been reported [20], [24], [25]. Furthermore, instrumentation for on-line monitoring allows for automated and data-rich experimentation. Crucially, microfabricated devices will allow thorough investigation of the effect of perfusion culture during process development with minimum use of expensive media [26], [27], [28], [29]. In larger reactors, perfusion cultures have shown improved expansion yields over static culture conditions for haematopoietic [30], [31] and embryonic stem cells [32], [33].

However, particular considerations must be made when designing a microfabricated bioreactor for regenerative medicine process development so that a link is maintained with conventional culture methods and production systems for the purposes of validation and scale up studies. Firstly, the hydrodynamic shear inherent in perfused systems can cause cell wash out of weakly adhering cells at flow rates as low as 0.05 ml/hr [34]. Furthermore, the effect of hydrodynamic shear may need to be decoupled from the effects of media replenishment and the removal of secreted factors. Secondly, dynamic seeding may result in non-uniform and poorly defined seeding densities, the presence of cells outside of the intended cell culture area, and damage to cells seeded in colonies (such as hESCs). Finally, the properties of the culture substrate and the extracellular matrix (ECM) affect cell adhesion, which in turn affects cell proliferation and cell differentiation. In current cell culture protocols, cell growth surfaces typically consist of a tissue culture polystyrene (TC-PS) culture substrate coated with an ECM. However, integration of TC-PS with microfabricated devices is difficult, since TC-PS is not compatible with conventional bonding and microfabrication techniques.

In this contribution, we start to address the above issues by presenting a novel, autoclavable, microfabricated culture device, with a re-sealable culture chamber. This re-sealable culture chamber allows traditional static seeding in an otherwise fully assembled device. Additionally, the device reversibly seals with a TC-PS microscope slide (or any other standard sized slide), allowing the use of traditional growth surfaces. Using computational fluid dynamics software, we analyse how hydrodynamic shear stress can be adjusted by recessing the cell culture area. We demonstrate the benefits of the device, by seeding feeder cells and hESC colonies in static conditions onto gelatine-coated TC-PS. We also demonstrate the use of low hydrodynamic shear stress perfusion in the culture of hESC colonies that maintain an undifferentiated morphology, and retain the expression of pluripotent markers under continuous perfusion culture. Finally, using a novel image processing algorithm, we show that hESC colonies can be detected against a background of feeder cells. In the future, this will allow real-time quantification of hESC colony sizes during cell culture.

## Results

### Microfabricated Modular Scale-down Device

**Figure 1:**
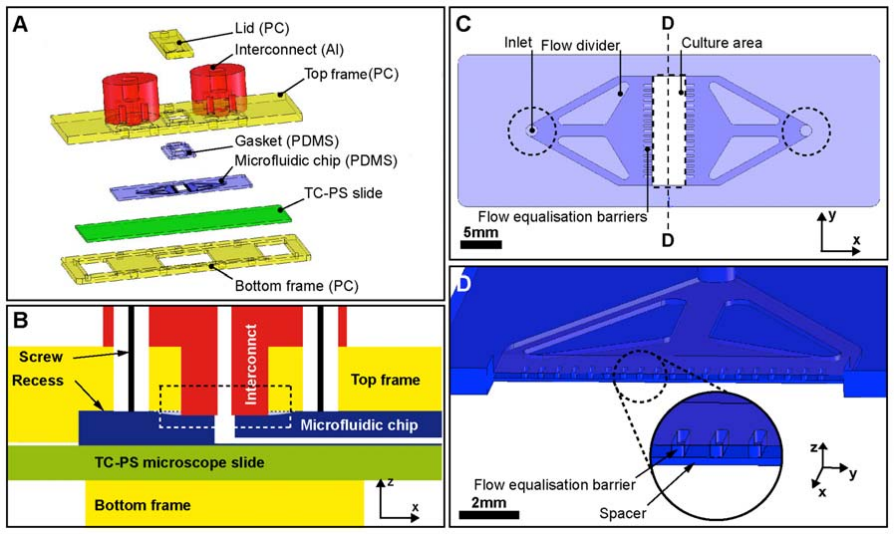
Design of the microfabricated culture device. (**a**) Exploded view showing all parts of the modular microfabricated culture device. (**b**) Schematic representation of a longitudinal section of the interconnect assembly, showing compression of the PDMS chip around the inlet/outlet ports (dashed rectangle), by the interconnect. (**c**) Top view of the microfluidic chip with dashed lines showing the footprints of the lid and interconnect bosses. (**d**) Cross-sectional view showing the two PDMS layers of the microfluidic chip. The lower ‘spacer’ layer elevates the flow equalisation barriers of the top layer and thus reduces the hydrodynamic shear exposure for the cells.

**Figure 2:**
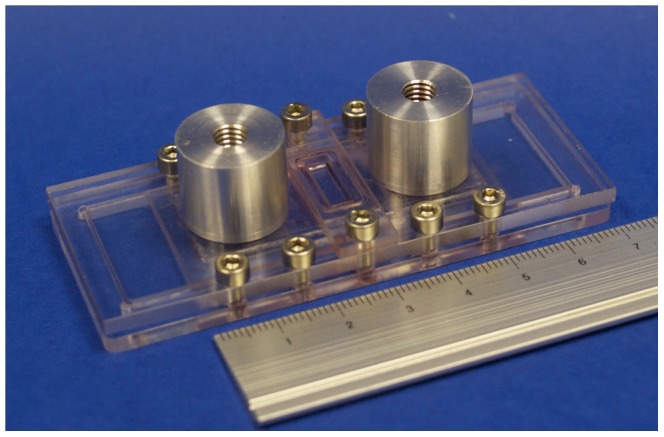
Photograph of the assembled modular culture device with the re-sealable lid attached.

The microfabricated culture device (Figures 1 and 2) consisted of a lid made from polycarbonate (PC), two interconnects made from aluminium (Al), a top and bottom frame (PC), a gasket and a microfluidic chip made from poly(dimethylsiloxane) (PDMS), and a TC-PS slide (16004, Nunc, Denmark).

The top frame included an opening to accommodate the lid as well as two recesses. The first positioned the microfluidic PDMS chip with respect to the top frame, and the second deeper recess accommodated the gasket. A set of bores in the top frame enabled the mounting of the two interconnects. The bottom frame had the same outer dimensions as the top frame and a recess dimensioned to hold the TC-PS slide. An opening in the centre was designed to bring objectives from an inverted microscope into close proximity with the TC-PS slide for cell culture imaging. The top and bottom frame were clamped together with five M3 hex screws distributed down each side of the frame. The central pair of screws also attached the lid when in use. All the screws were tightened to 2 N.cm forming seals between the components by compression of the PDMS.

To facilitate rapid set-up of cell culture experiments and achieve leak-free long-term operation, an easy and robust interconnection with the macro-world is required [35]. The cylindrically shaped interconnects (Figure 1(b)) contained a 1 mm diameter bore in their centre to link external tubing with the microfluidic chip. At the bottom, the interconnects formed a boss that compressed the microfluidic PDMS chip to form a seal. At the top, the bore was threaded to accept M6 Upchurch fittings and therefore permit simple connection with tubing for the provision and removal of media. The mean burst pressure of the culture device was 59 kPa with a standard deviation of 18 kPa (n = 36) and the lowest recorded burst pressure was 35 kPa. The pressure drop across the device at a flow rate of 500 ml.h^−1^ (3 orders of magnitude higher than the perfusion flow rate) was measured as 20 kPa.

The lid was T-shaped with the upper ‘horizontal’ bar acting as a bed stop when the lower ‘vertical’ bar was pushed into the opening of the top frame. This defined the height of the culture chamber below (450 µm). The ‘vertical’ bar formed a press-fit with the gasket to seal the chamber. The dimensions of the ‘vertical’ bar matched the footprint of the culture chamber of the microfluidic PDMS chip. The re-sealable lid provides a simple means to open and close the culture chamber. This enables operation of the device in a so-called ‘open’ configuration for cell seeding, and a ‘closed’ configuration for medium perfusion. Analysis of variance shows there is no statistically significant relationship between burst pressure and the number of times the lid is removed and reinserted for up to 30 repetitions (α = 0.05, p = 0.99, n = 3).

The PDMS microfluidic chip controls the flow of culture medium in the device. The microfluidic chip was made out of two PDMS layers with both containing a rectangular culture chamber measuring 4 mm in the direction of flow by 13 mm across the flow. The top layer (Figure 1(c)) contained the 200 µm deep flow channels connecting the inlet and outlet ports to the culture chamber. The flow is expanded from a narrow inlet prior to the culture chamber and condensed back to a narrow outlet after the chamber by 3 merging channels on each side. The top layer also included flow equalisation (or perfusion) barriers on each side of the culture chamber, each 200 µm wide and 1000 µm long. The apertures between the barriers itself had a rectangular cross-section (400 µm × 200 µm). The second layer (‘spacer’) elevated the first layer above the cell culture area by 120 µm (Figure 1(d)).

### Modelling Velocity Fields and Hydrodynamic Shear

**Figure 3:**
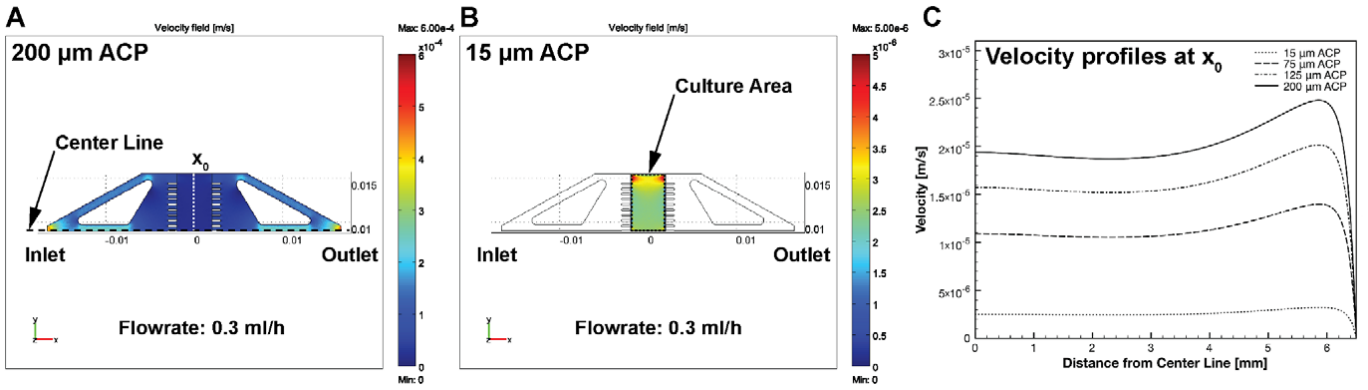
Modelling of flow conditions in the microfluidic chip. (**a**) represents the velocity field at half the height of the inlet channel. (**b**) represents the velocity field 15 um above the culture plane (ACP). (**c**) shows velocity profiles at x_0_ along the z-axis.

To evaluate the design, we analysed the velocity fields and shear stress produced at a flow rate of 300 µl.h^−1^. This flow rate corresponds to replacing 13.8 ml of media per day for each square centimetre of culture area, a rate 50 times higher than typical in hESC culture. It is therefore unlikely cells would ever be subjected to a higher shear stress. The uniformity of the velocity field in the culture device was investigated at various heights above the culture plane (i.e. above the TC-PS slide). 15 µm above the cell culture plane, the average fluid velocity is approximately a factor of 10 lower than at 200 µm above the cell culture plane, which is in line with the inlet and outlet channels (Figure 3 (a, b)). The microfluidic chip design produces a relatively even velocity field across the majority of the culture chamber (Figure 3(b, c)). An increased velocity at the boundaries of the culture chamber can be observed due to the larger gap between flow restrictor and the boundary. This effect was deliberate and intended to remove air bubbles, entrapped during closing or filling. Hydrodynamic shear stress was also calculated 15 µm above the cell culture plane for a flow rate of 300 µl.h^−1^. An average of 1.1×10^−4^ Pa and a standard deviation of 0.14×10^−4^ Pa were obtained from the model. The calculated value of 1.3×10^−4^ Pa, using an analytical solution for shear stress at the culture surface, supports the result obtained through finite element modelling.

### Static Seeding and Perfusion Culture of hESC Colonies

**Figure 4:**
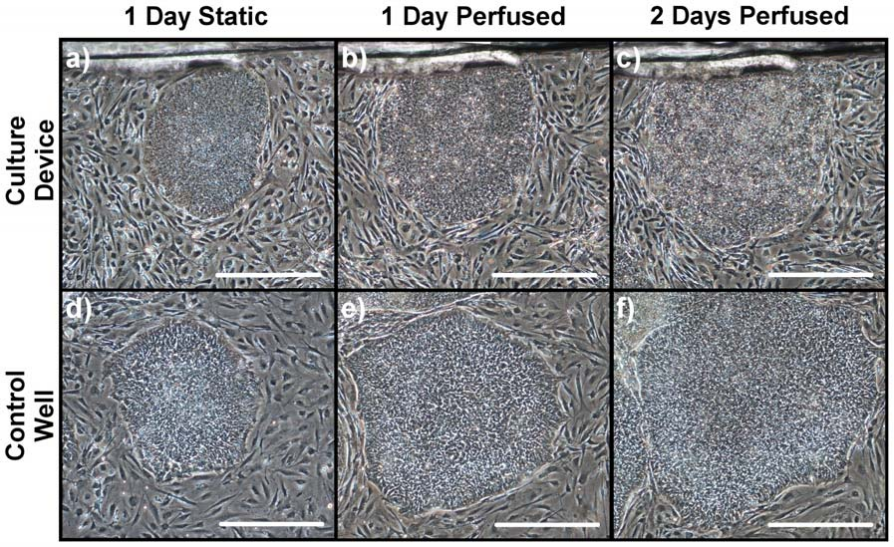
Co-cultured hESCs in microfabricated culture device and control dish. Representative phase contrast images of iMEF feeder cells and individual hESC colonies cultured in the microfabricated culture device (**a-c**) and in the control dishes (**d-f**). The same two colonies are shown at each of three time points; after 1 day of static culture (a, d), after 1 day of the perfused culture (b, e), and at the end of the 2 days of perfused culture (c, f). All images were taken with a 4× objective, scale bar is 500 µm.

To test the suitability of the device for hESC culture, we seeded culture devices, assembled from autoclaved parts, according to the protocol employed in our regenerative medicine laboratory (see Materials and Methods). As a control, we seeded three single-well dishes in parallel to the culture devices. In the culture devices and the control dishes, the inactivated mouse embryonic fibroblasts (iMEF) started to attach within 2 hours. After one day, the cells had attached and spread in both systems. hESC colonies seeded onto the iMEF layer attached within 1 day. Colonies maintained an undifferentiated morphology comparable to the colonies in the control dishes (Figure 4(a,d)).

A day after hESC seeding, the culture devices were closed and media was continuously perfused at 300 µl.h^−1^, resulting in a residence time of approximately 5 min. In the control dishes, the media was replaced once a day in line with standard manual cell culture practice. During perfusion, dissolved gases were supplied via the media having been absorbed from the incubator through the inlet tubing. After 1 day, the cells within the colonies were small and tightly packed together; a characteristic morphology of undifferentiated hESCs (Figure 4(b,e)). After 2 days of continuous perfusion, hESC colonies maintained an undifferentiated morphology in both the culture device and in the control dishes (Figure 4(c,f)). Representative higher magnification images are available in Supporting Information S1.

**Figure 5:**
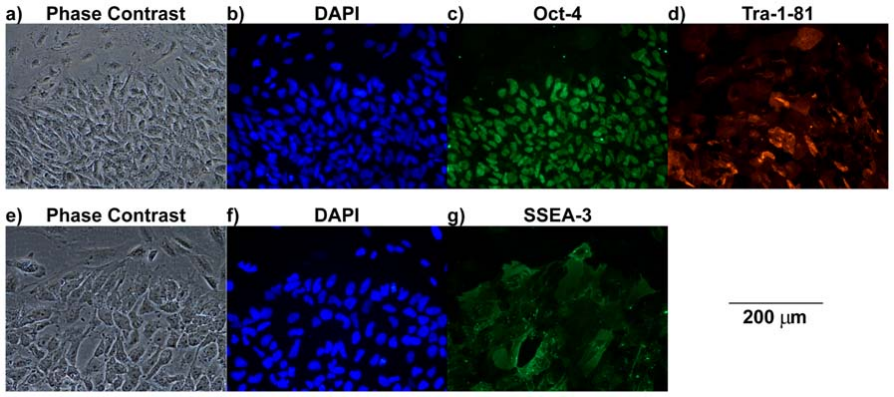
Staining of hESC colonies following perfusion culture. Representative images of the feeder cells and hESC colonies in the culture device after 2 days of continuous perfusion culture. Each row shows the phase contrast images (**a**, **e**) of the feeder-attached hESC colonies and the corresponding results from DAPI (**b**, **f**,) and pluripotency marker staining for Oct-4 (**c**), Tra-1-81 (**d**) and SSEA-3 (**g**). All images were taken with a 20× objective, scale bar is 200 µm.

Immunostaining was carried out to test for the expression of several pluripotency markers. The hESC colonies from one culture device were co-stained for Oct-4 and Tra-1-81, and the cells from a second device were stained for SSEA-3 (both were co-stained with DAPI). The immunostaining sequences of antibody incubation and washing buffers were performed in the device using the ‘open’ configuration, i.e. after removing the lid. As can be seen in figure 5, the hESC colonies stained positively for Oct-4 (c), Tra-1-81 (d) and SSEA-3 (g) with the correct localisation, nuclear, surface and surface respectively. The percentage of cells staining positive for Oct-4, in images of individual colonies, was 91% in the culture device and 94% in the control well with standard deviations of 2% and 5% respectively (n = 3 colonies, ∼1,500 cells total). In a repeat experiment, a culture device and a control dish were stained with Annexin V and propidium iodide (PI) to detect apoptotic and necrotic cells. The numbers of cells staining positive were very low (Supporting Information S2).

### Rapid Quantification of hESC Colony Size

**Figure 6:**
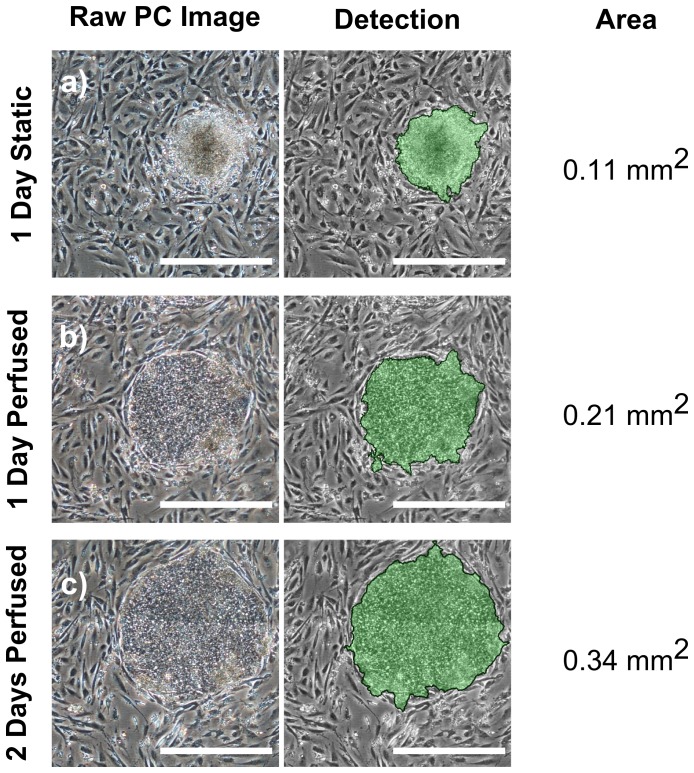
Monitoring a hESC colony in the microfabricated culture device during the course of an experiment. The same colony is shown after (a) 1 day static culture, (b) 1 day perfused culture and (c) 2 days perfused culture. The columns show, from left to right, the raw phase contrast image taken with a 4× objective, an overlay of the automated detection using the image processing algorithm, and the detected area. The scale bars are 500 µm.

An image processing algorithm was developed which permitted the detection of hESC colonies co-cultured with iMEF feeder cells. In brief, the texture of the local neighbourhood of a pixel was characterised at four scales (corresponding to various levels of spatial coarseness) and a random forest statistical classifier [36] used this information to label the pixel as being either part of a hESC colony or of the background (which included the iMEF cells). The resulting binary images can be used as a basis for the computation of the confluency (ratio of hESC pixels to total number of pixels) or the area occupied by the cells. This approach was used to monitor the culture in the microfabricated culture device. Figure 6 shows tracking of a single colony in the culture device from 1 day after seeding to the end of the 2 day perfusion period. The number of colonies, the total area occupied and the mean colony area were computed based on phase contrast images acquired at various stages of the expansion (Table 1). Differences in colony size and area can be attributed to the difference in the microenvironment of the cells. These include the medium exchange regime (perfusion vs static culture), the mass transfer, and variations from the manual dissecting of the colonies.

**Table 1:** Quantification of hESC colony size.

		Detected Colonies	Total Area (mm^2^)	Average Area (mm^2^)
**Culture Device**	Day 2	27	2.91	0.108
	Day 3	25	4.15	0.166
	Day 4	23	6.78	0.295
**Control Dish**	Day 2	17	2.28	0.134
	Day 3	16	5.04	0.315
	Day 4	10	8.20	0.820

Due to the time required to image larger culture areas, only the central area of the control dish, which contained the majority of colonies, was imaged. The numbers of distinct colonies shrinks as nearby colonies grow into each other.

Performance of the algorithm was evaluated against 20 unseen phase contrast images from the microfabricated culture device which showed a uniform range of confluencies from 3% to 82% (Supporting Information S3). To characterise performance we report F-scores, which are a standard metric to express the correctness of the pixel classification (and indicate overlap). The mean F-score was 90% with a standard deviation of 7% (n = 20), the worst F-score obtained over the testing set was 71%. In addition, the error in confluency estimation is assessed by comparing the confluency computed from the expert annotation and that derived from the algorithm output. The accuracy (bias) for the confluency estimates was −0.6% with a 95% confidence interval of [−2.1%, 1.0%]. The precision of the estimates, as determined from the root mean square error (RMSE) and the estimate bias was 3.2%. The algorithm takes up to 45 seconds for a high resolution image (1280×960). Additional metrics typically employed in image processing for pixel classification performance are reported in Supporting Information S4.

## Discussion

We present a microfabricated adherent culture device that starts to address the requirements of regenerative medicine process development and demonstrate the potential of the device by culturing feeder-attached hESC colonies. The culture of feeder-attached hESC colonies is an appropriate model system for multiple reasons. hESCs are more difficult to culture than other common model systems such as Chinese Hamster Ovary cells, mouse embryonic stem cells, mouse embryonic fibroblasts and human foreskin fibroblasts. Furthermore, hESCs are a clinically relevant cell type and co-culture techniques, which are inherently more complicated than monoculture, are common in regenerative medicine. Thus feeder-attached hESC culture is a more rigorous test than many other culture processes and it is assumed a device suitable for feeder-attached hESC culture would be suitable for most other adherent cell cultures.

### Design

Integration of TC-PS with microfluidic devices would normally be difficult, as TC-PS is not compatible with conventional air plasma or thermal bonding. Consequently, microfabricated devices for adherent cell culture make ubiquitous use of glass or poly(dimethylsiloxane) (PDMS) growth surfaces [37]; neither of which are commonly employed in regenerative medicine [38]. Indeed, to introduce novel growth surfaces and ECMs to processes for medical application, or even to compare them accurately to existing materials, would require extensive testing and validation. In our device, we successfully demonstrated the integration of gelatine-coated TC-PS through compression of the PDMS components against smooth surfaces resulting in an average burst pressure of 59 kPa. TC-PS is the most widely employed cell growth surface in stem cell biology, including T-flasks and Cell Factories, making later translation to larger production scales straightforward.

With our modular design, materials other than TC-PS can easily be integrated as long as they have a smooth, flat surface and the dimensions of a standard microscope slide [39]. This makes a number of materials immediately available for investigation. As a result, this device could be employed to test growth surface candidates from microarray screening [40], under the defined culture conditions obtainable in the microfluidic chip. This is analogous to the scale-up train in traditional biotechnology, where ‘hits’ from high-throughput screening plates are first investigated in shaker cultures or small-scale bioreactors.

The minimum size of the culture chamber is limited, not by the methods of microfabrication, but the number of cells required for analysis. We have designed our culture chamber to be as large as possible within the constraints of a microscope slide. The chamber was 13 mm wide and 4 mm in the direction of flow giving a culture area of 0.52 cm^2^ (between 96-well and 48-well plates). This is sufficient for immunostaining or quantitative PCR. Further, the form factor of the culture chamber must also be considered. When investigating the effects of specific process variables it is important that these variables are uniform across the entire cell culture area. However, in long, narrow perfusion chambers, the consumption and secretion of soluble factors by cells near the inlet alter the conditions for the cells downstream. This effect is exacerbated at lower flow rates. Consequently, when defining the culture area, the width dimension of the culture chamber was maximised, within the limits of the slide’s width, to minimise the length of the chamber. These dimensions are distinctly different from all other microfabricated devices for hESC culture [26], [27], [28], [41].

To promote uniformity across the culture chamber further, the top layer of the microfluidic chip (Figure 1(c, d)) included flow dividers and rows of flow equalisation barriers on either side of the culture chamber. The efficacy of flow equalisation barriers at creating uniform flow velocity fields was previously demonstrated with slightly larger apertures [42], and with smaller rectangular apertures [43]. We demonstrate the effectiveness in our design through the generation of a relatively uniform velocity field (Figure 3(c)). The barriers thus minimise non-uniform cell growth patterns which can arise from variations in velocity fields and which are difficult to interpret [44]. Such growth patterns could be caused by spatial differences in shear stress or spatial differences in the exchange of soluble factors.

### Seeding

Seeding density is a critical variable to both the expansion and differentiation of stem cell populations. Additionally, weakly adhering cells like hESC colonies typically require long incubation times (up to 2 days) to achieve secure attachment [45]. During this period, a culture medium overlay (typically a few millimetres) balances the oxygen and nutrient demands of the cells. Further, due to the low number and high value of some starting stem cell populations, a cell-efficient seeding method is required. Compared with dynamic seeding [46], static seeding gives more accurate control over starting cell density and distribution as it avoids cells settling and adhering in inlet and outlet channels. Additionally, the exposure to hydrodynamic shear stress occurring with flow-based, dynamic seeding methods is avoided. Minimising exposure to hydrodynamic shear is particularly crucial for the handling of embryonic stem cells, since shear stress during seeding can affect the phenotype [22] and could potentially dissociate multi-cellular hESC colonies. Finally, a device where standard seeding protocols can be adhered to reduces differences between scales and paves the way for robust and reproducible culture processes. We therefore sought to integrate a standard seeding method with our device to facilitate operation with a wide range of cells and seeding parameters.

**Figure 7:**
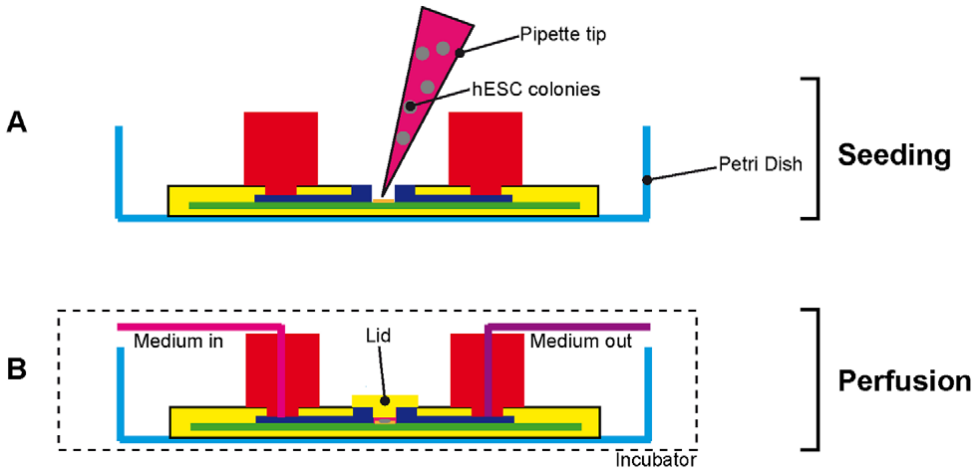
Schematic representation of a longitudinal section across the culture device. (**a**) Without a lid for coating with extra-cellular matrix compounds, and seeding feeder cells and hESC colonies with a pipette according to standard laboratory procedures. (**b**) With a lid and tubing for media perfusion.

To address this goal our device includes a re-sealable lid. The lid provides a simple means of opening and closing the culture chamber. Thus, the device can be operated on both open and closed configuration during culture protocols (Figure 7). In the closed configuration, the height of the culture chamber is repeatably defined by the re-sealable lid. Additionally, the hard material of the lid does not deform during medium perfusion ensuring reproducible fluid flow patterns. In the open configuration, the culture chamber is directly accessible with laboratory pipettes facilitating pipette-based methods typically employed in laboratory scale stem cell maintenance including static seeding, static cell recovery and immunostaining. A further advantage of our device is that, in open configuration, the depth of media in the culture chamber is similar to the depths used in T25-flasks or culture dishes. Thus, during cell settling and attachment, the cells experience a similar microenvironment to traditional culture systems, addressing our objective of maintaining a link to conventional culture methods for validation. Previously presented re-sealable systems required seeding before assembly [47], which is cumbersome and results in poorly defined culture areas, or limited the height of the culture chamber to the total thickness of the device [48], potentially leading to excessive media hold-up times during perfusion.

We successfully seeded both inactivated mouse embryonic fibroblasts (iMEF) and colonies of human embryonic stem cells (hESC) utilising standard static seeding protocols. The iMEF cells started to attach within 2 hours and had attached and uniformly spread after one day. hESC colonies attached to the uniform iMEF layer attached within 1 day and maintained an undifferentiated morphology comparable to control dishes (Figure 4(a, d)). Furthermore, there was no statistically significant relationship between repeated removal and reinsertion of the lid and the burst pressure of the device up to 30 iterations. These results confirm the suitability of the re-sealable lid in facilitating static cell seeding.

### Perfusion Culture

Previous reports indicated that shear stress is a critical parameter that can lead to cell dislodgement during medium perfusion [34], [49], which we confirmed in our own experiments (data not shown). The hydrodynamic shear stress of 1.1×10^−4^ Pa achieved in our design at 300 µl.h^−1^ is an order of magnitude below 5×10^−3^ Pa and three orders of magnitude below 1×10^−1^ Pa, the critical values previously reported by Korin *et al* [34] for hESCs and Toh et al. [29] for mESCs, respectively. Therefore, cell washout or significant shear impact are unlikely in our device. The low shear stress is primarily achieved through the large cross section of the culture chamber. However, shear stress on the culture plane is reduced further by recessing it below the inlet and outlet channels. The effectiveness of this technique has been previously demonstrated in straight channels with grooves [49], round wells [48] and rectangular chambers [42]. In our design, the PDMS ‘spacer’ layer (Figure 1(d)) elevates the main plane of medium flow above the cell growth surface. Since the thickness of the layer is determined by spin-coating parameters, the elevation can easily be changed. An example application is the optimisation of shear stress levels at a fixed flow rate or vice versa.

Supporting our predictions from fluid dynamic modelling, we did not observe washout of hESC colonies at the relatively high flow rate of 300 µl.h^−1^. We successfully demonstrated a 2-day, continuous perfusion culture of feeder-attached hESC colonies in a microfluidic device without washout of the colonies. Both the low shear chip design and the use of traditional substrate may have contributed to the continued adherence and growth of the cells in these conditions. While these results must be further verified with feeder cell densities that match more closely the densities from the control dishes, the results demonstrate the suitability of the microfabricated device as a culture system for hESCs. Further, the lack of infection after 3 days of culture, along with additional *E. Coli* clearance studies (Supporting Information S5, S6), demonstrates the effectiveness of sterilisation by autoclave. Finally, the positive staining results, in combination with the morphology observations, are evidence supporting a maintained, undifferentiated hESC state during seeding and continuous perfusion.

### Monitoring

Adherent cell cultures are by nature difficult to monitor: whereas suspension cultures can be characterised by sampling small culture volumes for offline analysis or by using indirect cell density measurements such as optical density, no standard approach is readily available for adherent systems. However, to accelerate regenerative medicine bioprocessing, there is clearly a need for a quantitative method for online characterisation of adherent cell cultures in general and that of co-cultures in particular. Such an online characterisation method will allow accurate and reproducible measurement of the effect of changes in experimental conditions (e.g. culture substrate and ECM used, medium formulation). To this effect, an image processing approach was developed to automate the detection and characterisation of hESC colonies co-cultured with iMEF feeder cells (Figure 6) without the addition of dyes or markers to the culture medium.

Conventional microscopy image processing methods, which are based on the detection of local changes in intensity are unable to distinguish between two cell populations as they present similar intensity profiles. Instead, our approach relies on the detection of differences in texture between hESC colonies and fibroblast cells. The random forest classifier is essentially a set of complex rules that in this case are used to label each of the pixels according to their texture features. Using information from the neighbourhood of a pixel at multiple scales is necessary for a robust characterisation of texture. The process mimicked how a human expert would distinguish the two cell types by evaluating multiple features in local regions of the image. The algorithm achieved a high pixel classification performance, which resulted in a low confluency estimation error. Our confluency estimates were shown to have no significant bias (mean = −0.6%, 95% CI = [−2.1%, +1.0%]), and a precision of 3.2%; together these show that it produces estimates in good agreement with that of a human expert.

Some of the discrepancies in detection results can be attributed to limitations of the current algorithm or to inadequate human annotations. Indeed, it is often challenging to classify pixels in ambiguous regions of the image such as colony borders or dense iMEF clusters. However, the random forest classifier was chosen to alleviate issues with ambiguous choices and the results reported here-in demonstrated the versatility and the accuracy of the approach. Furthermore, we show the algorithm can be used to generate metrics of colony number and size. Combined with the low computational complexity of the algorithm, this makes the method suitable for on-line monitoring of hESC culture confluency. To process the 18 4× images required to cover our culture chamber takes less than 15 min.

As a subject for future studies, culture results with adult stem cells need to be developed to address the full scope of regenerative medicine. Furthermore, the current study could be strengthened by the addition of further online and end-point measurements, including *in vivo* functional evaluation of the cells expanded in this device. This would enhance comparison with other more conventional methods of stem cell expansion. We are currently integrating further monitoring capabilities, such as the detection of bulk and peri-cellular dissolved oxygen concentrations, and building a fully automated parallelised platform that fits on a microscope stage. This will permit real-time data-rich experimentation for regenerative medicine process development.

## Materials and Methods

### Fabrication of the Culture Device

All parts and moulds were designed with a 3D CAD system (SolidWorks 2007, Dassault Systemes SolidWorks, USA). Aluminium parts were machined by conventional CNC machining. Polycarbonate (PC) parts were fabricated from PC sheets (3 mm, 681-637, RS, UK, and 5 mm, 681-659, RS, UK) with a CNC micro-milling machine (M3400E, Folken Industries, USA) using 2-flute standard length end mills (Kyocera Micro Tools, USA). The G-code for the micro-milling machine was directly generated from the CAD files (MasterCam X2, CNC Software, USA). Structured PDMS (Sylgard 184, Dow Corning, USA) parts were cast in moulds milled out of 5 mm thick poly(methylmethacrylate) (PMMA) (20070, Nordisk Plast, Denmark), and 3 mm thick aluminium. The moulds were inspected with an SEM (XB1540 “Cross-Beam”, Carl Zeiss AG, Germany). A schematic of the fabrication steps and SEM images of the mould can be found in Supporting Information S7, S8. Unstructured layers of PDMS were fabricated by spin-coating (P6708D, Specialty Coating Systems, USA) PDMS on a silanised (85041C, Sigma-Aldrich, UK) 4″ silicon wafer (Prolog Semicor, Ukraine) and cured at 80°C for 1 hour. To bond PDMS parts they were rinsed with ethanol, dried, and bonded using an air plasma (90 s, 30 W, 500 mTorr, PDC-002, Harrick Plasma, USA), and cured in an oven at 80°C for 2 hours.

### Burst Pressure Measurements

To measure burst pressure a 10 ml plastic syringe was connected to one interconnect via tubing and a 3-way valve (98-2750, Harvard Apparatus, UK). Tubing connected to the other interconnect was blocked with a Luer lock plug. The third port of the 3-way valve was connected to a pressure sensor (40PC100G, Honeywell, USA) glued into a fitting (P-207, Upchurch Scientific, USA) with epoxy glue. A syringe drive was used to pump air into the device at 5 ml.min^−1^ and the pressure was logged via a LabView™ routine (LabView 2011, National Instruments, USA) and data acquisition card (USB-6229BNC, National Instruments, USA). The burst pressure was taken as the highest recorded applied pressure for a given experiment. The burst pressure was recorded 3 times per assembly for 12 different assemblies. Additionally, for the last 3 assemblies, single burst pressure measurements were made following iterations of lid removal and reinsertion. Measurements were made following 1–10, 20 and 30 iterations. Lid replacement burst pressures were normalised against the initial burst pressure before applying analysis of variance to investigate a relationship between burst pressure and lid replacement. All device components used for burst pressure experiments had previously been autoclaved.

### Fluid Dynamic Modelling

The Navier-Stokes equations were solved by using the finite element method (FEM) software package Comsol Multiphysics 3.5a (COMSOL, Cambridge, UK). A fully developed steady-state flow with no slip condition at the boundaries was assumed. Water at 37°C was used as working fluid with interpolated values for density and dynamic viscosity of 993.2 kg.m^−3^ and 6.96×10^−4^ Pa.s, respectively [50]. The boundary conditions were set at the inlet to an average velocity calculated from the flow rate (300 µl.h^−1^), and at the outlet to zero pressure. Due to the longitudinal symmetry of the microfluidic chip, only half of the chip was incorporated in the model to minimise computational time. Tetrahedral elements were employed to mesh the 3-D domains of the culture device (mesh sizes between 2.5 to 7.5 µm, 527539 elements). The model was solved with a built-in linear system solver UMFPACK.

Hydrodynamic shear stress was calculated from the simulated velocity profile using the equation.
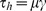
where *τ_h_* is the shear stress at a height *h* from the surface, *μ* the dynamic viscosity and γ the shear rate.

To verify and compare the calculated shear stress from the model, the analytical solution of the equation for the wall shear stress between infinite parallel plates was used:
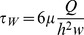

*τ_w_* is the shear stress at the wall, *h* the height of the culture chamber, *w* the width of the culture chamber, *μ* the dynamic viscosity and *Q* the volumetric flow rate.

### Ethics Statement

Mouse embryonic fibroblasts (MEFs) were derived from mouse embryos, which were harvested at day 12.5–13.5 of pregnancy (E12.5-13.5) from a naturally mated CD-1 female mouse. The pregnant female and the embryos were humanely sacrificed following Schedule 1 of the Animals (Scientific Procedures) Act 1986, for which specific ethical approval and licence are not required according to UK regulations.

### Cell Maintenance

Shef-3 hESC line (<passage 70) obtained from the UK Stem Cell Bank were cultured on a layer of feeder cells. Primary mouse embryonic fibroblasts (MEFs) (<passage 5) were used as a feeder layer. They were maintained in Dulbecco’s Modified Eagle Medium (DMEM) (41965, Invitrogen, USA) supplemented with 10% (v/v) heat inactivated foetal bovine serum (FBS) (10270, Invitrogen, USA) and 1% (v/v) Modified Eagle Medium Non-Essential Amino Acids (MEM NEAA) (11140, Invitrogen, USA), passaged every 3 days into T25-flasks (159910, Nunc, Denmark) and cultivated in a humidified incubator at 37°C and 5% CO_2_. To inactivate the MEFs the growth medium was replaced with 5–7 ml of normal MEF growth medium supplemented with 1 mg.ml^−1^ of mitomycin C (M4287, Sigma-Aldrich, UK) and incubated for 2 hours at 37°C. After inactivation cells were washed three times with Dulbecco’s phosphate buffer solution (DPBS) (D1408, Sigma-Aldrich, UK), detached by incubating with a trypsin:EDTA solution (T4049, Sigma-Aldrich, UK) for 3 minutes, quenched with normal MEF medium, centrifuged and re-suspended in MEF media. Cells were seeded at a density of 9,200 cells cm^−2^ into T25-flasks that had been pre-coated with a 0.1% (w/v) in DPBS gelatine solution (G1890, Sigma-Aldrich, UK) for 10 minutes at room temperature.

The hESCs were cultivated in KnockOut DMEM (10829, Invitrogen, USA) supplemented with 20% KnockOut Serum Replacement (10828, Invitrogen, USA), supplemented with 1% MEM NEAA (11140, Invitrogen, USA), 2 mM L-Glutamine (21051, Invitrogen, USA), 0.1 mM β-mercaptoethanol (M3148, Sigma-Aldrich, UK) and 4 ng.ml^−1^ FGF2 (4114-TC, R&D Systems, USA). For passaging, flasks were incubated with 1.5 ml of 0.025 mg.ml^−1^ collagenase solution (17104, Invitrogen, USA) for 5 minutes, before being replaced with fresh hESC medium. hESC colonies were then dissected into medium-sized colonies using pasteur pipettes and transferred into a new flask containing feeder cells prepared as outlined above.

### Cell Culture

Prior to each cell culture experiment, all parts of the culture device and all tubing and tools required for assembly were autoclaved. The culture device was then assembled with a sterile TC-PS slide in a laminar flow hood. For substrate coating and cell seeding, the lid was removed (‘open’ configuration, Figure 7(a)). Laboratory pipettes with 200 µl pipette tips were employed for all steps. The TC-PS surface of the culture chamber and three single-well dishes (353653, BD Biosciences, USA; culture area 2.89 cm^2^) were coated with 0.1% (w/v) gelatine in DPBS solution, and left to incubate at room temperature for 15 min. Then, each dish was seeded with ∼45,000 inactivated MEFs (seeding density of ∼15,600 cells cm^−2^) in 1000 µl of MEF medium. The culture device was seeded with ∼15,000 inactivated MEFs (∼ 28,800 cells cm^−2^) in 200 µl of MEF medium. (A higher cell density was chosen for the culture device to ensure confluency.) The dish and culture device were transferred to incubator (37°C, 5% CO_2_). For transfer between laminar flow hood and incubator, the culture device was placed in a large sterile glass Petri Dish (2175553, Schott, USA). 1 day later the MEF media was replaced with hESC media, dissected hESC colonies were seeded in the culture device and the control dishes, and both were incubated (37°C, 5% CO_2_) for a further day.

**Figure 8:**
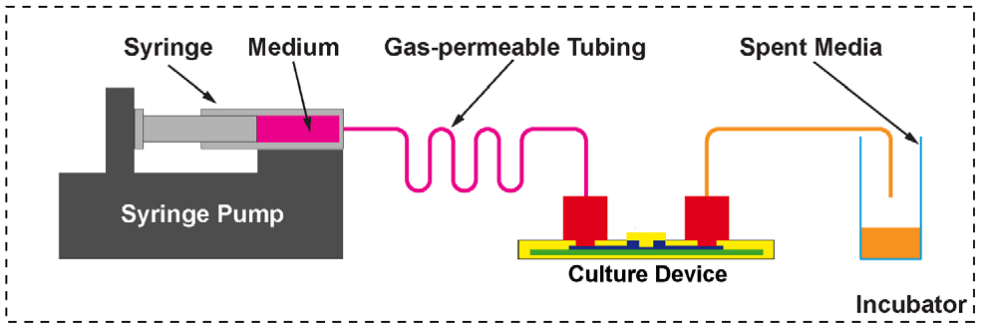
Schematic representation of the continuous media perfusion setup. A syringe pump is used to pump media through gas-permeable tubing to adjust gaseous tension levels before entering the culture device.

After 1 day of static culture, the medium in the culture chamber was aspirated and the culture device closed with the re-sealable lid (‘closed’ configuration, Figure 7(b)). An autoclavable tubing (R1230, Upchurch Scientific, USA) with Upchurch fittings (P207, Upchurch Scientific, USA) and a gas-permeable silastic tubing (R3607, Tygon, USA), connected the syringe with the culture device (Figure 8). The two types of tubing were attached to each other via Luer adapters (F331 and P659, Upchurch Scientific, USA). The gas permeable tubing was included to adjust gaseous tension levels in the media before entering the culture chamber. The culture device was manually primed with culture medium using a syringe after which, the syringe was placed on a syringe drive (Model100, KD Scientific, USA) and culture medium perfused for 2 days at 300 µl.h^−1^. The entire setup was placed in an incubator to maintain the culture temperature and atmospheric composition. Medium in the control dishes was exchanged every day.

### Cell Staining and Imaging

Daily cell culture inspections and end-point assay imaging were performed with an inverted microscope (Eclipse TE2000-U, microscope camera DS-Fi1, Nikon, Japan). Cell staining in the culture device was performed in the open configuration. For apoptosis/necrosis staining, cells were washed once with DPBS then incubated for 5 min with Annexin V-FITC and propidium iodide (PI) each diluted 1∶100 in binding buffer (K101-25, BioVision, USA). For immunostaining, hESC colonies were fixed with 4% (v/v) paraformaldehyde (PFA) in phosphate buffered saline (PBS) for 20 minutes then washed three times. All washing was with PBS supplemented with 10% (v/v) FBS to block non-specific binding. Cells to be stained for nuclear marker Oct-4 were permeabilised by incubating with 0.2% Triton X-100 for 15 min at room temperature before washing a further 3 times. We incubated cells with primary monoclonal antibodies Oct-4 (SC-5279, Santa Cruz, USA) or SSEA-3 (ab109868, Abcam, UK), at a dilution of 1∶200 in blocking solution, for one hour at room temperature. The cells were then washed three times and incubated with secondary antibodies that had an excitation wavelength of 488 nm (A11017, Invitrogen, USA and A21212, Invitrogen, USA respectively) for one hour at room temperature. Cells stained for Oct-4 were then washed three times and co-stained for Tra-1-81 by repeating the primary/secondary staining procedure above with a Tra-1-81 primary (ab16289, Abcam, UK) and a secondary with an excitation wavelength of 555 nm (A21426, Invitrogen, USA). Finally, the cells were washed with DPBS and stained with 4',6-diamidino-2-phenylindole (DAPI) (D1306, Invitrogen, Carlsbad, CA, USA). DAPI at a dilution of 1∶200 was incubated with cells at room temperature for 10 minutes. Three experts counted cells staining positive for DAPI and Oct-4 in images of individual and partial colonies. Cells in images of three different colonies were counted in both the culture device and the control dish. The average counts across the three users were used to calculate the percentage of positive cells in each colony image.

### Automated hESC Colonies Characterisation

All image processing was done using MATLAB (version R2011a, MathWorks Inc., USA) and 1280×960 images taken using a 4× objective. Images were first converted to a double-precision floating point greyscale representation by computing a weighted average of the three image channels (weighted 0.290, 0.570 and 0.140 for red, green and blue components respectively). The basic image features (BIFs) of the image were computed from the responses of derivative-of-Gaussian filters according to the scheme of Crosier and Griffin [51]. Briefly, the BIF approach classifies each pixel of the image into one of seven classes based on approximate symmetry in its neighbourhood. The parameter *σ* defines the scale of the derivative-of-Gaussian filters employed. The readiness of the algorithm to ignore local structure and classify a pixel as ‘flat’ is controlled by the parameter ε. BIFs were computed at scales *σ_base_*, 2*σ_base_*, 4*σ_base_* and 8*σ_base_* (*σ_base_* = 0.7), with *ε* kept constant at 0.11. For each scale, a local histogram of the counts of different BIFs appearing in a 25×25 pixels uniformly weighted window was built for each pixel of the image. The resulting features vector for each pixel contained 28 elements (4 concatenated local BIF histograms of 7 bins each). A MATLAB implementation of the random forest classifier [52] was trained using 1.52×10^7^ pixels annotated by a human expert. Each pixel was labelled as either hESC or background (which also included fibroblast cells). The random forest consisted of 20 trees with 5 variables randomly sampled at each split.

When processing an image, the features associated with each pixel were computed as outlined above and the random forest classifier was used to predict the class labels. The result was a binary image with hESC pixels equal to 1 and the rest to 0. Finally, small objects were discarded as detection noise (size <4000 pixels) and holes were filled (size <6000 pixels) using binary morphological operations.

Detection performance was evaluated by comparing the output of the image processing algorithm to results of a human expert. The testing set included 20 representative images (cropped to 500×500 pixels each) of typical hESC cultures in the microfabricated culture device at different time points. These images were independent from those used for training. The F-score was computed as following:
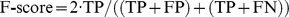
where *TP* was the number of true positives, *FP* the number of false positives, and FN the number of false negatives. The confluency was computed as the ratio of the number of pixels set to 1 (hESC pixels) to the total number of pixels. The area of detected colonies was computed by multiplying the number of pixels set to 1 by a calibration factor relating pixels to distance (for a 4× lens, at a resolution of 1280×960 pixels, 1 pixel was equal to 2.86 µm^2^). See Supporting Information S4 for the full set of pixel classification metrics.

## Supporting Information

Supporting Information S1 **Representative higher magnification phase contrast images of hESC colonies in the culture device.** Phase contrast images of hESC colonies after (a) 1 day of static culture and (b) 1 and (c) 2 days of perfused culture in the microfabricated culture device. All images were taken with a 10× objective, scale bar is 200 µm.
(TIF)

Supporting Information S2 **Images from viability staining of hESC colonies following perfusion culture.** Images of a hESC colony after 2 days perfused culture in the microfabricated culture device. From left to right (a) a phase contrast image taken after staining, (b) annexin V staining and (c) PI staining. All images were taken with a 20× objective, scale bar is 200 µm.
(TIF)

Supporting Information S3 **Testing set of 20 images.** For each image, the panel on the left shows the border detected by the image processing algorithm in blue overlaid on the grayscale phase contrast image. The panel on the right shows the details of the detection with the true positives in yellow, the true negatives in black, the false positives in green, and the false negatives in red. The scale bar is 500 µm.
(TIF)

Supporting Information S4 **Evaluation of pixel classification performance.** Algorithm outputs for 20 representative hESC images were compared to human expert annotations resulting in the performance metrics listed.
(DOCX)

Supporting Information S5 **Samples of broth from** ***E. Coli*** **clearance test.** Two PDMS chips and two PC lids were incubated for 17 hours at 37°C in Terrific Broth containing *E. Coli* XL10-Gold Kan^r^ (Stratagene, UK). One of each type of part was then autoclaved before each of the four parts were placed in separate shake flasks of sterile Terrific Broth and incubated on a shaker for 6 hours along with a flask containing only media (negative control). This figure shows samples of broth from each flask below their respective OD_600_ measurements. From left to right; autoclaved PDMS chip, autoclaved PC lid, positive control PC lid, positive control PDMS chip, negative control.
(TIF)

Supporting Information S6 **Agar plates from** ***E. Coli*** **clearance test.** Agar plates showing zero colony forming units following seeding of 100 ml of broth incubated with the (a) PDMS and (b) PC parts respectively (see Supporting Information S5) and a 1 day incubation at 37°C. Significant growth occurred in positive controls (data not shown).
(TIF)

Supporting Information S7 **Fabrication process of a mould and a microfluidic chip.** (1) A sheet of Dural® was machined with a micromilling machine to create a mould (2). (3) PDMS was cast into the mould and then degassed. A PC sheet was placed on top of the mould to clamp the mould. Concurrently, a silanised silicon wafer was spin coated with PDMS to form a membrane. The PDMS-coated wafer and the clamped mould were then cured for 1 hour at 80°C in an oven. (4) The microfluidic manifold layer was released from the mould and the culture chamber body was cut out. (5) The microfluidic manifold layer and the PDMS membrane were exposed to an air plasma and immediately brought into contact for bonding. (6) The membrane at the bottom of the culture chamber body was cut out and the microfluidic chip was cut in shape and released from the wafer. Schematic representation is not to scale.
(TIF)

Supporting Information S8 **Scanning Electron Microscopy images of the mould for the microfluidic chip.** The negative flow equalisation barriers were milled with a 200 µm end mill (**a**). Burrs were not observed at the edges of the mould, for example at the edges of the flow equalisation barriers (**b**).
(TIF)
