# Supplementary figures and images for: Microfabricated Modular Scale-Down Device for Regenerative Medicine Process Development
Source: PLoS One. 2012 Dec 19;7(12):e52246. doi: 10.1371/journal.pone.0052246 (PMC3526573; doi:10.1371/journal.pone.0052246)

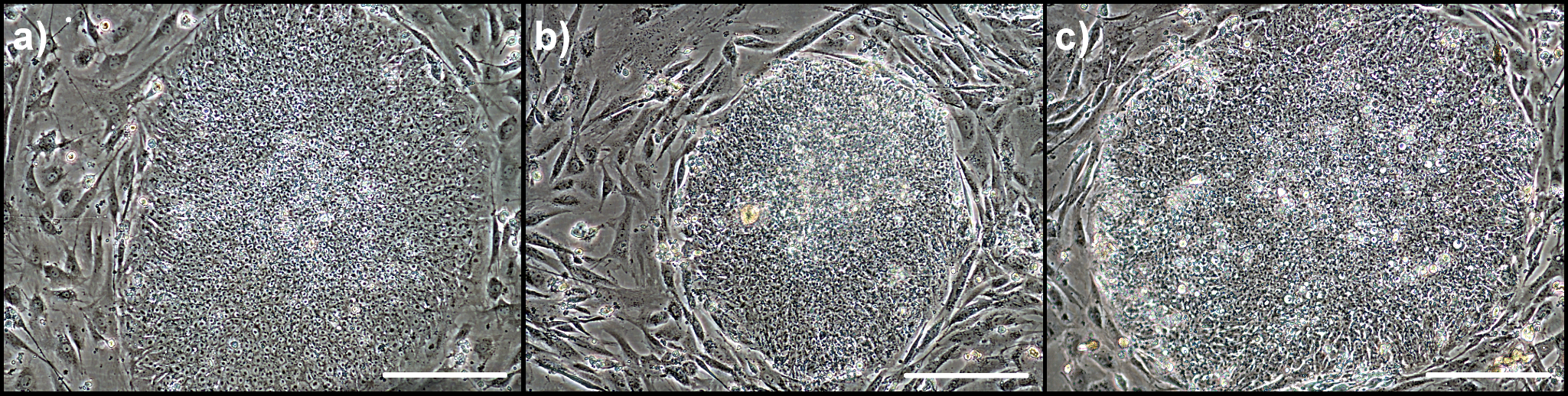

Supplement: Supporting Information S1 — Representative higher magnification phase contrast images of hESC colonies in the culture device. Phase contrast images of hESC colonies after (a) 1 day of static culture and (b) 1 and (c) 2 days of perfused culture in the microfabricated culture device. All images were taken with a 10× objective, scale bar is 200 µm. (TIF) [file pone.0052246.s001.tif]

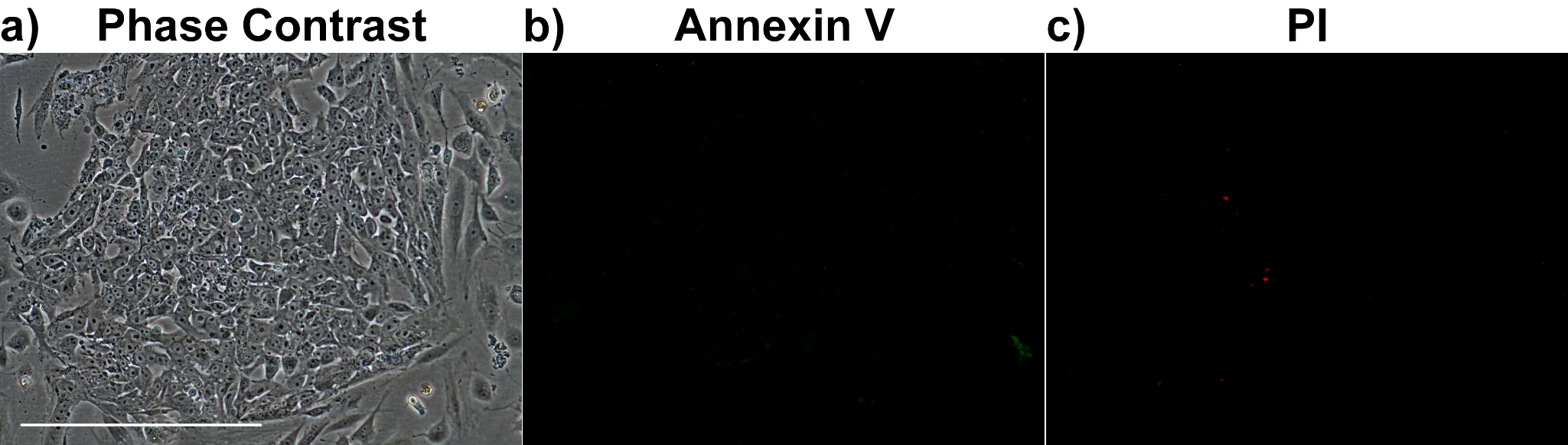

Supplement: Supporting Information S2 — Images from viability staining of hESC colonies following perfusion culture. Images of a hESC colony after 2 days perfused culture in the microfabricated culture device. From left to right (a) a phase contrast image taken after staining, (b) annexin V staining and (c) PI staining. All images were taken with a 20× objective, scale bar is 200 µm. (TIF) [file pone.0052246.s002.tif]

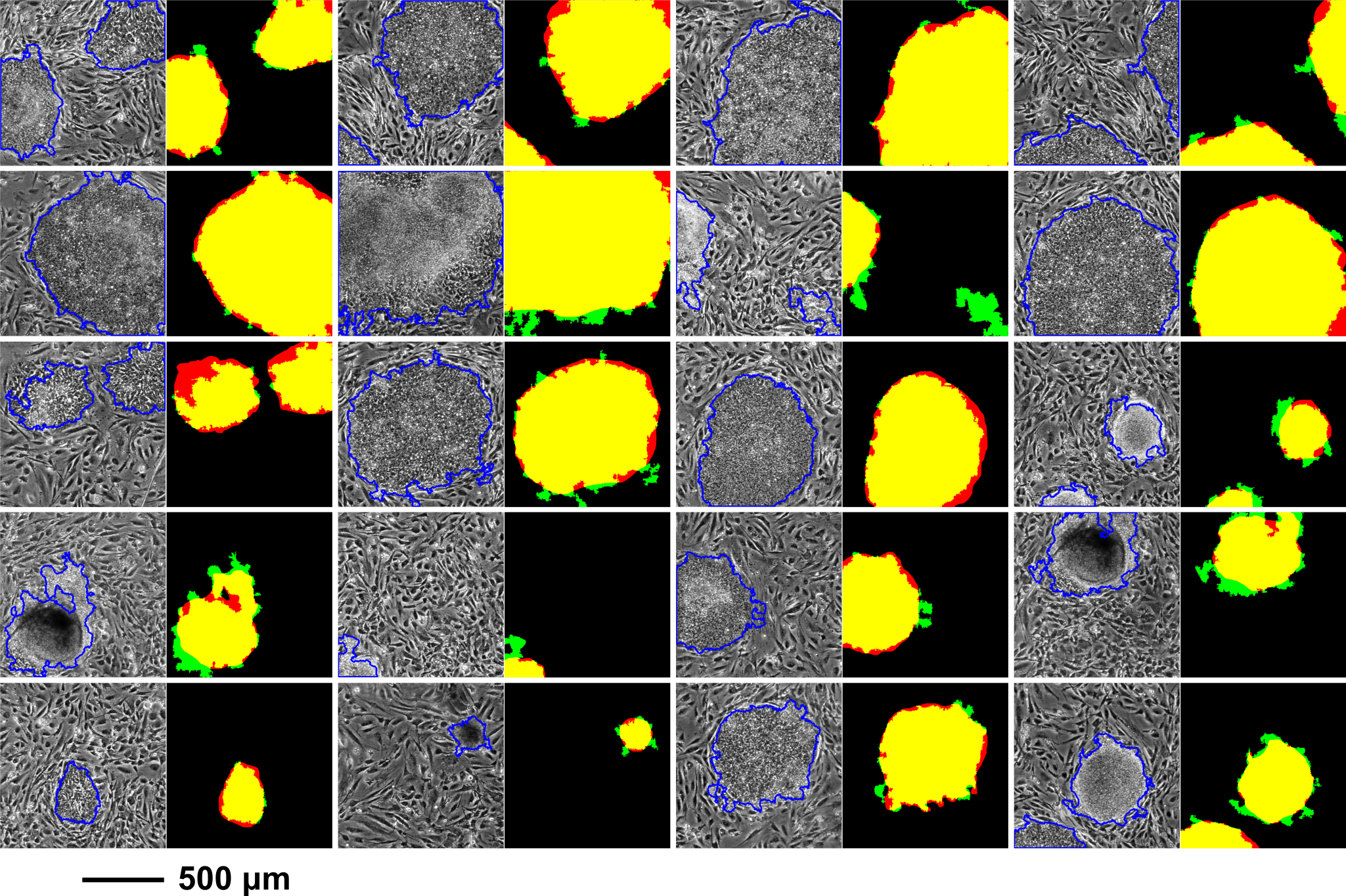

Supplement: Supporting Information S3 — Testing set of 20 images. For each image, the panel on the left shows the border detected by the image processing algorithm in blue overlaid on the grayscale phase contrast image. The panel on the right shows the details of the detection with the true positives in yellow, the true negatives in black, the false positives in green, and the false negatives in red. The scale bar is 500 µm. (TIF) [file pone.0052246.s003.tif]

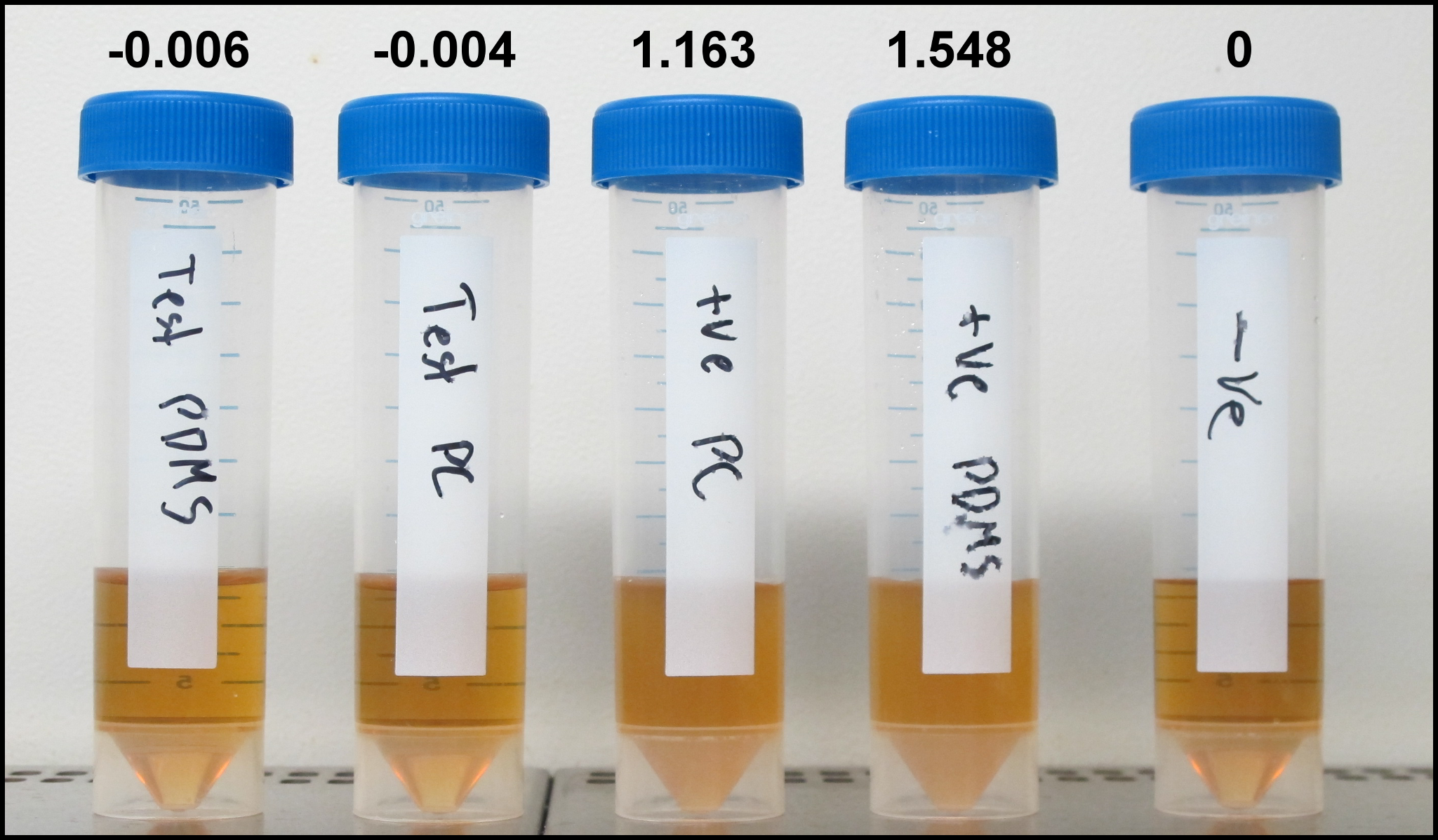

Supplement: Supporting Information S5 — Samples of broth from E. Coli clearance test. Two PDMS chips and two PC lids were incubated for 17 hours at 37°C in Terrific Broth containing E. Coli XL10-Gold Kanr (Stratagene, UK). One of each type of part was then autoclaved before each of the four parts were placed in separate shake flasks of sterile Terrific Broth and incubated on a shaker for 6 hours along with a flask containing only media (negative control). This figure shows samples of broth from each flask below their respective OD600 measurements. From left to right; autoclaved PDMS chip, autoclaved PC lid, positive control PC lid, positive control PDMS chip, negative control. (TIF) [file pone.0052246.s005.tif]

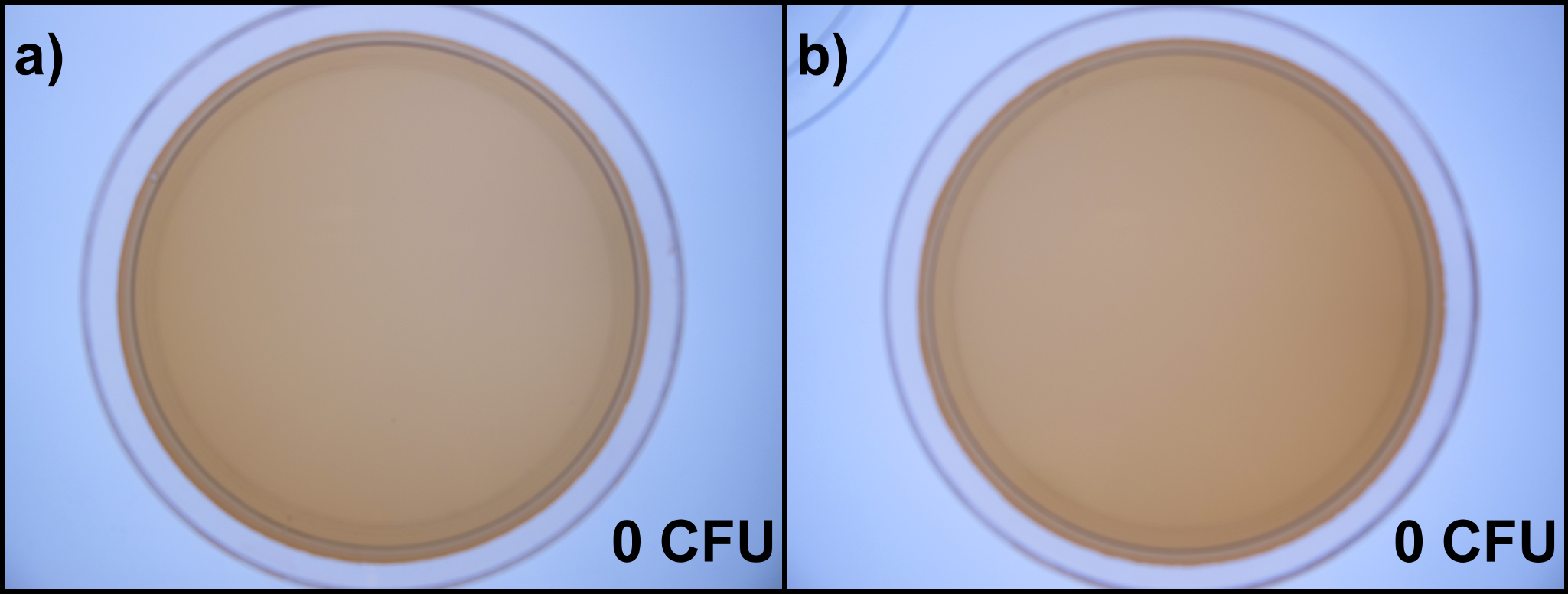

Supplement: Supporting Information S6 — Agar plates from E. Coli clearance test. Agar plates showing zero colony forming units following seeding of 100 ml of broth incubated with the (a) PDMS and (b) PC parts respectively (see Supporting Information S5) and a 1 day incubation at 37°C. Significant growth occurred in positive controls (data not shown). (TIF) [file pone.0052246.s006.tif]

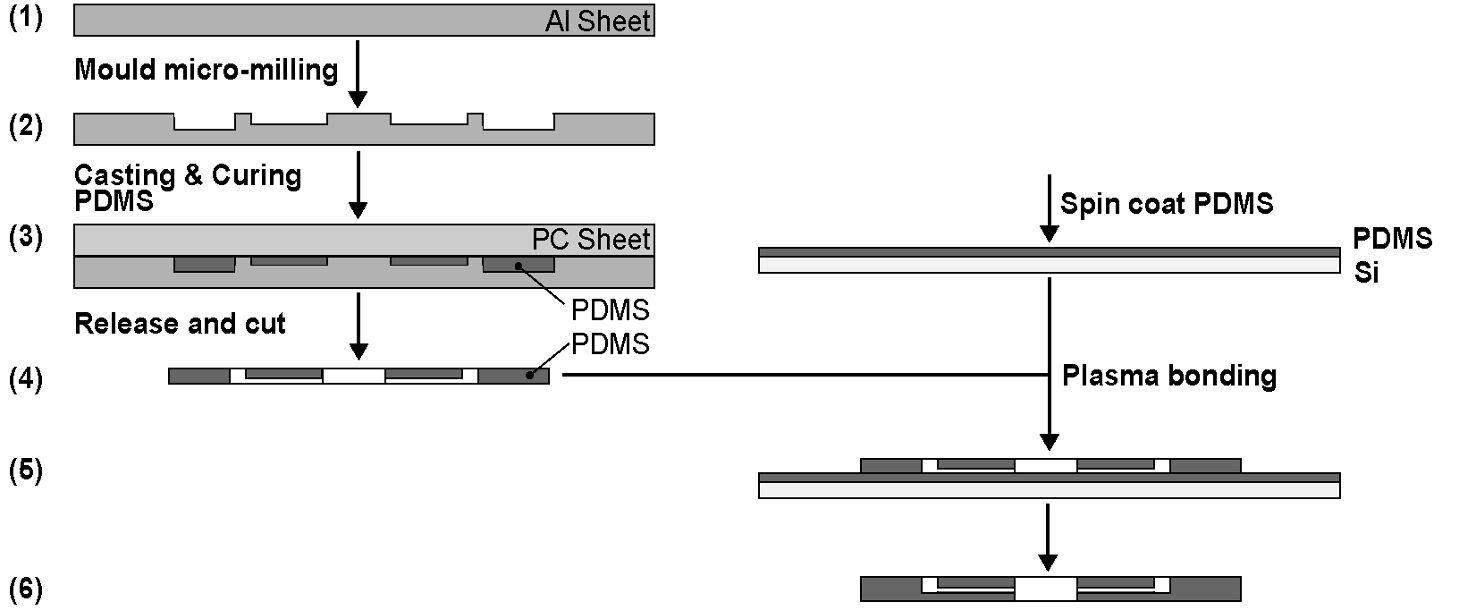

Supplement: Supporting Information S7 — Fabrication process of a mould and a microfluidic chip. (1) A sheet of Dural® was machined with a micromilling machine to create a mould (2). (3) PDMS was cast into the mould and then degassed. A PC sheet was placed on top of the mould to clamp the mould. Concurrently, a silanised silicon wafer was spin coated with PDMS to form a membrane. The PDMS-coated wafer and the clamped mould were then cured for 1 hour at 80°C in an oven. (4) The microfluidic manifold layer was released from the mould and the culture chamber body was cut out. (5) The microfluidic manifold layer and the PDMS membrane were exposed to an air plasma and immediately brought into contact for bonding. (6) The membrane at the bottom of the culture chamber body was cut out and the microfluidic chip was cut in shape and released from the wafer. Schematic representation is not to scale. (TIF) [file pone.0052246.s007.tif]

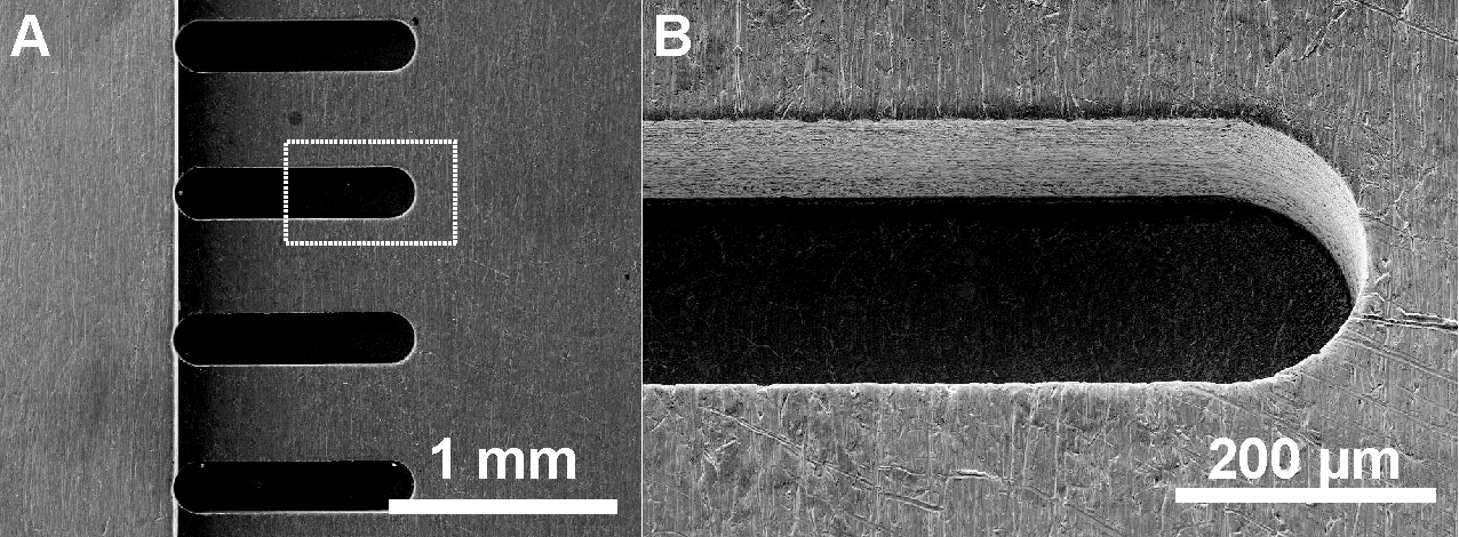

Supplement: Supporting Information S8 — Scanning Electron Microscopy images of the mould for the microfluidic chip. The negative flow equalisation barriers were milled with a 200 µm end mill (a). Burrs were not observed at the edges of the mould, for example at the edges of the flow equalisation barriers (b). (TIF) [file pone.0052246.s008.tif]
