# Supplementary material for: Microfabricated Modular Scale-Down Device for Regenerative Medicine Process Development
Source: PLoS One. 2012 Dec 19;7(12):e52246. doi: 10.1371/journal.pone.0052246 (PMC3526573; doi:10.1371/journal.pone.0052246)
Supplement: Supporting Information S4 — Evaluation of pixel classification performance. Algorithm outputs for 20 representative hESC images were compared to human expert annotations resulting in the performance metrics listed. (DOCX) [file pone.0052246.s004.docx]

| **Metric** | **Definition** | **Mean** | **Standard deviation** | **95% CI** |
| --- | --- | --- | --- | --- |
| F-scores | 2.TP/((TP + FP) + (TP + FN)) | 0.900 | 0.072 | [0.866,0.933] |
| Precision | TP/(TP + FP) | 0.897 | 0.121 | [0.840,0.953] |
| Recall/Sensitivity | TP/(TP + FN) | 0.914 | 0.051 | [0.890,0.938] |
| Accuracy | (TP + TN)/((TP + FN) + (FP + TN)) | 0.955 | 0.019 | [0.946,0.965] |
